# SUMO polymeric chains are involved in nuclear foci formation and chromatin organization in *Trypanosoma brucei* procyclic forms

**DOI:** 10.1371/journal.pone.0193528

**Published:** 2018-02-23

**Authors:** Paula Ana Iribarren, Lucía Ayelén Di Marzio, María Agustina Berazategui, Javier Gerardo De Gaudenzi, Vanina Eder Alvarez

**Affiliations:** Instituto de Investigaciones Biotecnológicas Dr. Rodolfo A. Ugalde—Instituto Tecnológico de Chascomús (IIB-INTECH), Universidad Nacional de San Martín (UNSAM)—Consejo Nacional de Investigaciones Científicas y Técnicas (CONICET). Campus Miguelete, Av. 25 de Mayo y Francia, San Martín, Buenos Aires, Argentina; University of Hull, UNITED KINGDOM

## Abstract

SUMOylation is a post-translational modification conserved in eukaryotic organisms that involves the covalent attachment of the small ubiquitin-like protein SUMO to internal lysine residues in target proteins. This tag usually alters the interaction surface of the modified protein and can be translated into changes in its biological activity, stability or subcellular localization, among other possible outputs. SUMO can be attached as a single moiety or as SUMO polymers in case there are internal acceptor sites in SUMO itself. These chains have been shown to be important for proteasomal degradation as well as for the formation of subnuclear structures such as the synaptonemal complex in *Saccharomyces cerevisiae* or promyelocytic leukemia nuclear bodies in mammals. In this work, we have examined SUMO chain formation in the protozoan parasite *Trypanosoma brucei*. Using a recently developed bacterial strain engineered to produce SUMOylated proteins we confirmed the ability of *Tb*SUMO to form polymers and determined the type of linkage using site-directed mutational analysis. By generating transgenic procyclic parasites unable to form chains we demonstrated that although not essential for normal growth, SUMO polymerization determines the localization of the modified proteins in the nucleus. In addition, FISH analysis of telomeres showed a differential positioning depending on the polySUMOylation abilities of the cells. Thus, our observations suggest that *Tb*SUMO chains might play a role in establishing interaction platforms contributing to chromatin organization.

## Introduction

SUMOylation is a eukaryotic post-translational modification that involves the covalent attachment of a Small Ubiquitin-like Modifier (SUMO) to a diverse range of protein substrates. SUMO conjugation is analogous to ubiquitination, and requires the sequential action of activating (E1), conjugating (E2) and eventually ligating (E3) enzymes [1, 2]. This enzymatic cascade catalyzes the formation of an isopeptide bond between the carboxyl group of the C-terminal Gly residue in SUMO and the ε-amino group of a lysine residue in the substrate protein. The target lysine residue is often embedded within a canonical consensus motif, ΨKxE (where Ψ is a hydrophobic residue and x any amino acid) [3] or in related variants encompassing an adjacent cluster of negative charges [4–6] recognized by the E2 enzyme[7]. In addition, SUMOylation can take place at non-consensus sites [8], but in these cases the process is usually assisted by E3 ligases.

The main consequence of protein SUMOylation is an alteration of the interaction surface where SUMO can recruit effector proteins containing short stretches of hydrophobic residues known as SUMO-interacting motifs (SIMs) [9, 10]. This modification can then be translated into changes in the subcellular localization, the subset of interacting partners, and the stability, among other outputs [1, 2]. Protein SUMOylation can be reverted by the action of specific peptidases [11] contributing to a dynamic modification of the biochemical properties of the target proteins.

Proteomic studies have highlighted the role of SUMO in many central cellular processes, such as DNA replication and repair, chromatin organization, transcriptional control, RNA processing, transport, macromolecular assembly and signal transduction [12, 13]. Accordingly, SUMO has been shown to be essential for the viability of many eukaryotic cells [14–17].

Trypanosomes, unicellular organisms which belong to a diverse group of eukaryotes of the excavate lineage [18], possess a single SUMO gene [16, 19, 20]. These protozoan parasites have a major impact on human and animal health. *Trypanosoma cruzi* is the causative agent of Chagas disease in South America while *Trypanosoma brucei* is the ethiological agent of African sleeping sickness in humans and nagana in cattle. *T*. *brucei* is a model organism amenable for genetic manipulation and knock-down experiments demonstrated that *Tb*SUMO is essential for mitosis in both procyclic (PCF) and bloodstream (BSF) forms of the parasite, which are the replicative forms present in the tsetse fly vector and the infected mammal, respectively [16, 17]. Moreover, site-specific proteomic studies of PCF identified a number of SUMOylated proteins involved in fundamental nuclear biological processes [21]. Interestingly, SUMO has been linked to *T*. *brucei* antigenic variation, a process where the major surface antigenic protein is replaced by a different variant with certain frequency as a strategy to elude the specific immune response of the host. *Tb*SUMO was found to be enriched in a particular region of the nucleus of BSF parasites together with the E3 ligase Siz1 and the RNA polymerase I, specifically at the chromatin region that is actively transcribing the variant surface glycoprotein (VSG), suggesting that a highly SUMOylated focus provides an environment permissive for *VSG* transcription [22].

Although in most cases SUMO is conjugated as a monomer, this modifier also shares with ubiquitin the ability to form chains. Mammalian SUMO-2/-3 isoforms [23] as well as yeast SUMO (Smt3) [24, 25] bear internal SUMOylation sites at their N-terminal regions that allow polymerization, and from the phenotype of SUMO chain mutants the contribution of the polymeric signal has started to be appreciated. In particular, structural roles for poly-SUMO chains in the formation of the synaptonemal complex [26] or promyelocytic leukemia nuclear bodies [27] have been described.

In this work, we have investigated SUMO chain formation in *T*. *brucei*. Using an *in bacteria* SUMOylation system, we confirmed the ability of *Tb*SUMO to polymerize and identified the site of branching using site-directed mutagenesis. By generating transgenic PCF parasites unable to form chains we demonstrated that although not essential for normal growth, these polymers are involved in the assembly of nuclear foci, likely establishing interaction platforms in the nucleus, which might contribute to chromatin organization.

## Results

### Lysine 27 is required for *Tb*SUMO chain formation

**Fig 1:**
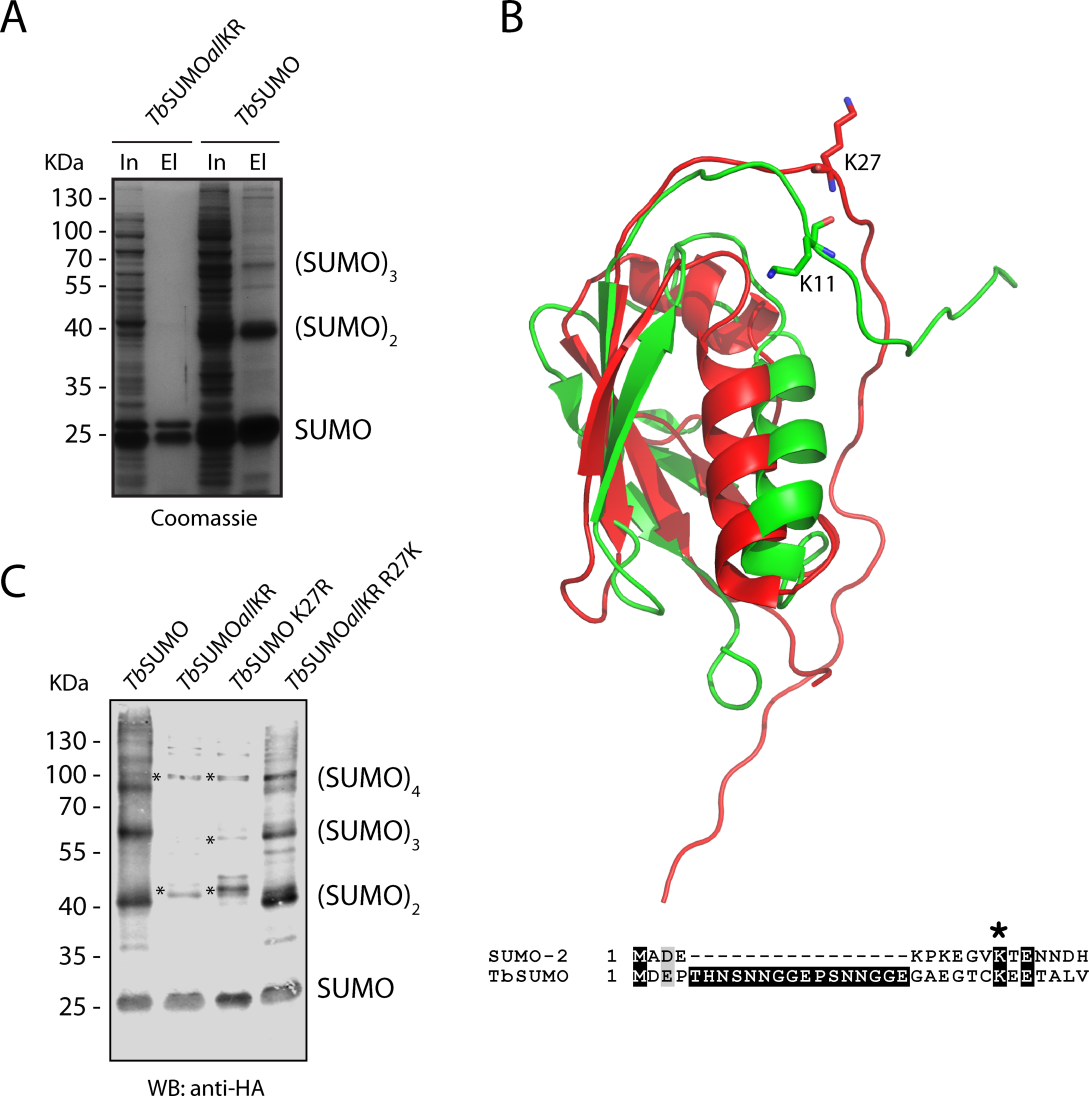
*Tb*SUMO chain formation *in bacteria*. **(A)** *E*. *coli* BL21 DE3 cells were used to co-express the complete SUMOylation system using either a wild type *Tb*SUMO variant (*Tb*SUMO) or a mutant version with all lysine residues replaced by arginines (*Tb*SUMO*all*KR). Cleared cell lysates were subjected to Ni^+2^ affinity chromatography and input (In) and eluates (El) were analyzed by 10% SDS-PAGE followed by Coomassie Blue staining. The positions of *Tb*SUMO monomer (SUMO), dimers (SUMO)_2_ and trimers (SUMO)_3_ are indicated. Note that the E2 enzyme (upper band of the doublet in the lane that corresponds to the eluate from *Tb*SUMO*all*KR strain) copurifies with SUMO (S1 Fig). **(B)** Comparison of the three dimensional structure of *Tb*SUMO (2K8H, red) and human SUMO-2 (2N1W, green). Sequence alignment of N-terminal extensions reveals a potential conserved SUMOylation site in *Tb*SUMO K27 marked with an asterisk. **(C)** *In bacteria* SUMOylation assays were performed with different HA-tagged *Tb*SUMO variants. Cleared lysates were subjected to Ni^+2^ affinity chromatography and analysed by Western blot using anti-HA antibodies. Note that in addition to SUMO polymers, the bands marked with asterisks could also correspond to SUMOylated forms of other components of *T*. *brucei* SUMOylation machinery, such as *Tb*E2 or *Tb*E1a (S1 Fig).

To facilitate the biochemical validation of *T*. *brucei* SUMO targets, we have previously developed an *in bacteria* SUMOylation system consisting of the heterologous co-expression of the SUMOylation machinery of the parasite (*Tb*E1a/E1b, *Tb*E2, *Tb*SUMO), together with a potential substrate, in *E*. *coli* [28]. When testing this system in the absence of any substrate, we detected a ladder of wild-type SUMO (*Tb*SUMO) that was no longer visible when all eight lysine residues present in the protein were mutated to arginine (*Tb*SUMO*all*KR) (Fig 1A). This observation led us to speculate that *Tb*SUMO, similar to its human counterparts SUMO-2/-3 or yeast Smt3, is capable of forming poly-SUMO chains.

Inspection of the amino acid sequence within the unstructured N-terminal region (Fig 1B) revealed that the lysine residue in position 27 (K27) could be the branching site, being predicted to be SUMOylated by different bioinformatic tools (GPS-SUMO [29], JASSA [30], SUMOAMVR [31], PCI-SUMO [32]). To establish the importance of K27 in the formation of poly-SUMO chains, this residue was changed to arginine (K27R), which preserves the charge on the molecule but cannot be engaged in the formation of an isopeptide bond. After *in bacteria* SUMOylation, reaction products were analysed by Western blot (Fig 1C). Whereas wild type *Tb*SUMO formed multimers, these higher molecular weight forms were absent when the assays were performed with *Tb*SUMO*all*KR or *Tb*SUMO K27R. Furthermore, the pattern of *Tb*SUMO*all*KR was reverted to that of wild type SUMO when K27 was restored (*Tb*SUMO*all*KR R27K). Thus, we conclude that poly-SUMO chains are mainly formed through K27 linkages.

### *Tb*SUMO chains bind to SUMO-interacting motifs placed in tandem

**Fig 2:**
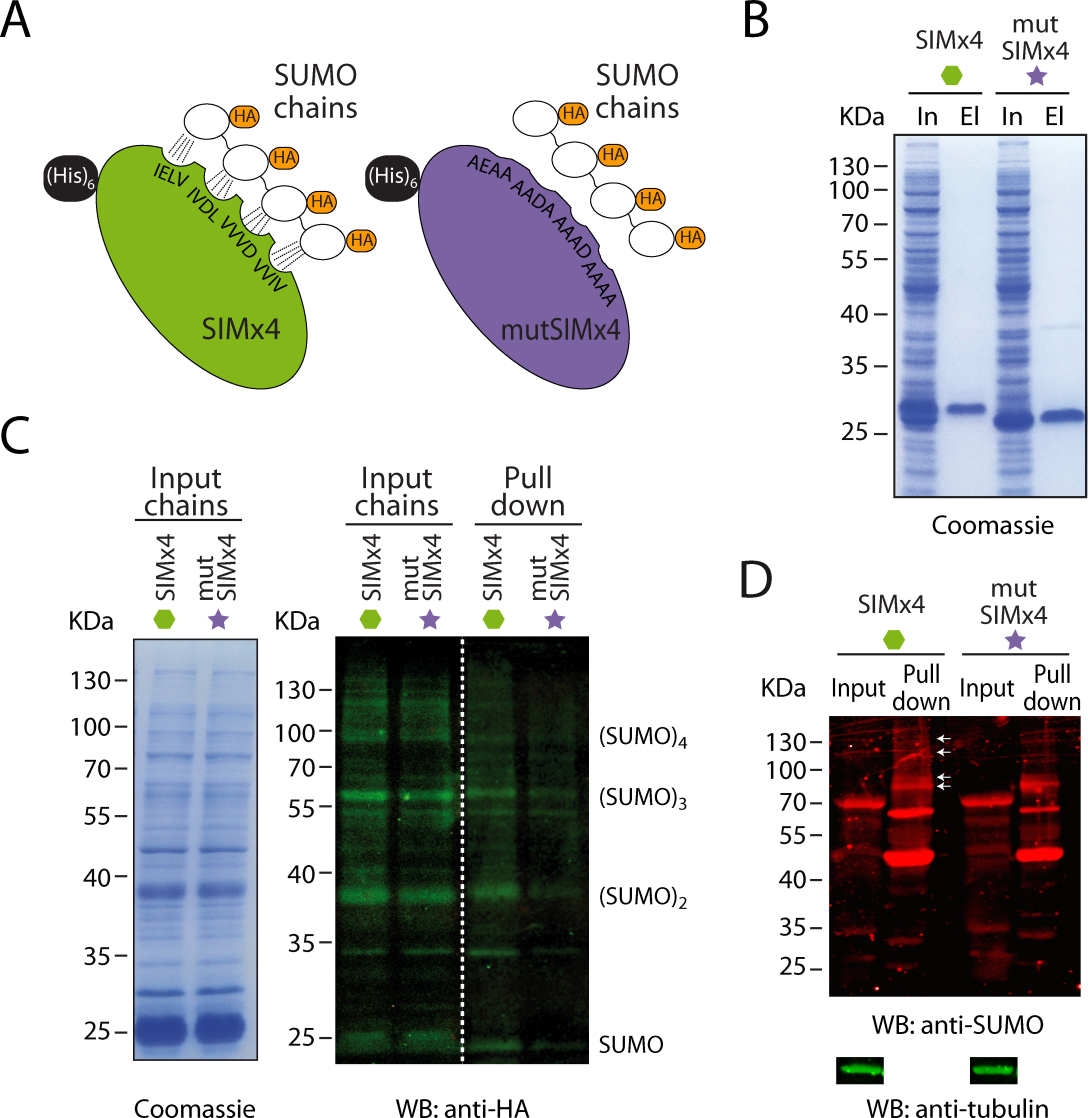
*TbS*UMO-SIM binding assays. **(A)** Illustration of the domain arrangement in SIMx4 and mutSIMx4 probes. **(B)** Coomassie Blue stained SDS-PAGE analysis of probe purifications. *E*. *coli* BL21 DE3 cells expressing SIMx4 or mutSIMx4 probes were lysed and cleared lysates were subjected to Ni^+2^ chromatography. Inputs, In; Eluates, El. **(C)** *E*. *coli* BL21 DE3 cells expressing HA-*Tb*SUMO chains (Inputs) were incubated with the SIMx4 or the mutSIMx4 probes. Samples were pulled-down using Ni^+2^-Sepharose beads. Proteins were visualized by Western blot using anti-HA antibodies. Coomassie stained gels of the inputs are shown. **(D)** Cell free extract from *T*. *brucei* 427 PCF was incubated with SIMx4 or mutSIMx4 probes, which were subsequently purified by Ni^+2^ chromatography. The capture of SUMOylated proteins was determined by Western blot analysis using anti-*Tb*SUMO antibodies, while anti-α-tubulin was used as the loading control for inputs.

Having shown that *Tb*SUMO is able to form chains, we next examined if they display the typical ability of binding to SUMO-interacting motifs (SIMs) placed in tandem. To test this, we used a probe originally developed for SUMO-2 [33] consisting of a fragment of the Ring Finger Protein 4 (RNF4) comprising four SIMs (SIMx4) and a mutated version in which Val, Leu and Ile residues within SIMs were changed to Ala (mutSIMx4) to disrupt SUMO-SIM interaction [10] (Fig 2A and 2B). Protein extracts expressing HA-*Tb*SUMO monomer (monomer) or polySUMO(chains) were incubated separately with SIMx4, mutSIMx4 or Ni^+2^-Sepharose beads without adding SIMx4 or mutSIMx4 probes (negative control), and their capacity to recognize both probes was tested (Fig 2C and S2 Fig). After washing and elution, Western blot analysis with anti-HA antibodies was performed for three replicates. The results showed that SIMx4 probe was able to pull-down *Tb*SUMO chains with higher ability (more than 3-fold increase) than the mutSIMx4 probe or Ni^+2^-Sepharose beads alone (Fig 2C and S2B Fig). However, under identical experimental conditions the SUMO monomer bound with relatively similar capacity to any of the bait tested (SIMx4, mutSIMx4 or beads) since no statistical differences were observed in the amount of protein detected in the eluates (S2C and S2D Fig). Taking together, the previous results indicate that poly-SUMO chains produced in bacterial systems are capable of binding to SIMs placed in tandem.

After this biochemical validation we used the probe with cell-free extracts from *T*. *brucei* 427 PCF, and incubated equal parts in parallel with either purified SIMx4, mutSIMx4 or beads alone (Fig 2D and S2E Fig). To preserve the modification, these experiments were performed with N-ethylmaleimide (NEM), a well-known inhibitor of SUMO deconjugating proteases and Western blot analysis with anti-*Tb*SUMO antibodies was performed. Although quantification of proteins bands in the eluates for three replicates revealed no statistical differences between poly-SUMO conjugates bound to SIMx4 in comparison to mutSIMx4 (S2F Fig), some high molecular weight bands were mildly enriched in the experiment pulled-down with SIMx4 (see arrows in Fig 2D).

### Generation of SUMO chain mutant parasites

**Fig 3:**
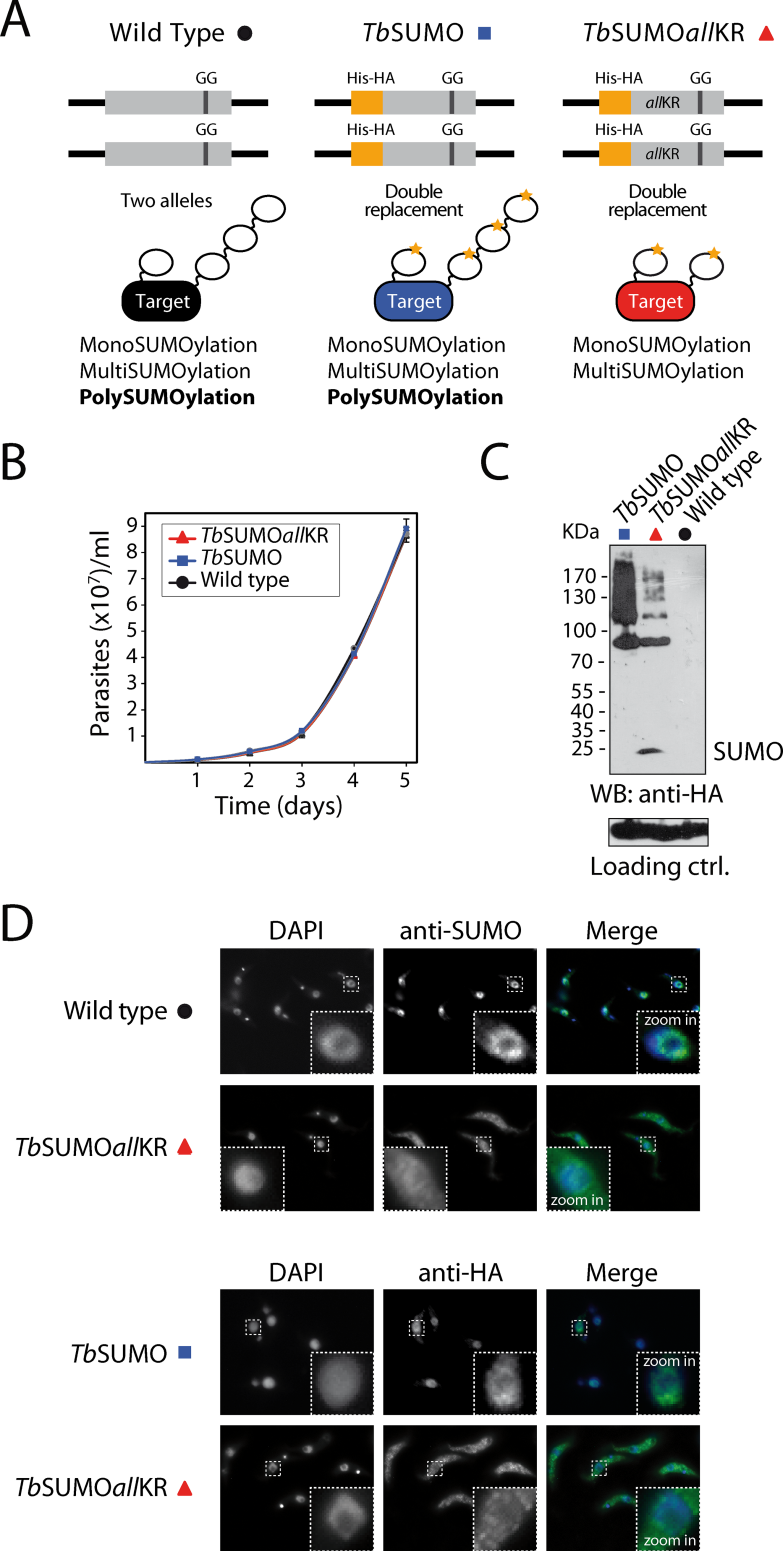
SUMO chain mutant *T*. *brucei* procyclic parasites. **(A)** Schematic representation of the SUMO variants expressed in PCF parasites. GG, diglycine motif essential for SUMO processing, activation and conjugation. **(B)** Growth of HisHA-*Tb*SUMO*all*KR strain (*Tb*SUMO*all*KR) compared to HisHA-*Tb*SUMO (*Tb*SUMO) and wild-type parasites. Wild-type and transgenic parasites were cultured up to one month without observing significant differences in growth rate. **(C)** Conjugating capacity of HisHA-*Tb*SUMO (*Tb*SUMO) and HisHA-*Tb*SUMO*all*KR (*Tb*SUMO*all*KR) parasites. Whole-cell extracts were boiled in Laemmli sample buffer immediately after harvesting, separated in a 7.5%-12.5% discontinuous acrylamide gel (3,5x10^7^ cells/lane), and analysed by Western blot using anti-HA antibodies. An unspecific cross-reacting band of ∼50 kDa from anti-*Tc*SUMO antibodies [22] was used as loading control. **(D)** Immunofluorescence analysis of wild-type, HisHA-*Tb*SUMO (*Tb*SUMO) and HisHA-*Tb*SUMO*all*KR (*Tb*SUMO*all*KR) parasites. Nuclear and kinetoplast DNA were visualized by DAPI staining (blue). Representative images of anti-*Tb*SUMO (green), anti-HA (green) and *Tb*SUMO/anti-HA-DAPI merged images are shown.

To study the biological roles of SUMO chains in *T*. *brucei*, we generated mutant PCF parasites in which the endogenous SUMO gene was replaced by the open reading frame of *Tb*SUMO where all eight lysine codons were mutated to code for arginine. As described above, the resulting protein can be conjugated to one or more sites of a target (mono or multi-SUMOylation), but lacks the ability to form chains (polySUMOylation) due to the absence of internal modification sites (Fig 3A). The SUMO variant expressed by these parasites is an unprocessed *Tb*SUMO version with an N-terminal His-HA tag (HisHA-*Tb*SUMO*all*KR). For comparison, a His-HA tagged *Tb*SUMO keeping all internal lysine residues was also generated (HisHA-*Tb*SUMO).

After two rounds of transfection and sequential replacement of both alleles by homologous recombination (*T*. *brucei* is diploid), parasites were cloned by limiting dilution and mutations were confirmed by DNA sequencing (S3 Fig). Unlike *Tb*SUMO-deficient cells, which arrest in G2/M [16], HisHA-*Tb*SUMO*all*KR strains are viable; indicating that the proteins modified with SUMO*all*KR can fulfill the essential functions for the parasite, at least under regular culture conditions (Fig 3B).

Comparison of the SUMOylation pattern between His-HA-tagged *Tb*SUMO variants able or unable to form chains by Western blot using anti-HA antibodies evidenced an increase in free (unconjugated) SUMO protein, and a decrease in the intensity of high molecular weight SUMO conjugates (Fig 3C) likely reflecting the absence of polySUMOylated proteins in the *Tb*SUMO*all*KR mutant.

To further investigate potential differences we evaluated the subcellular localization of *Tb*SUMO in wild type, HisHA-*Tb*SUMO and HisHA-*Tb*SUMO*all*KR parasites. In wild type PCF as well as in HisHA-*Tb*SUMO parasites, *Tb*SUMO accumulates in discrete foci dispersed in the nucleus (Fig 3D). This characteristic localization agrees with the fact that most SUMO targets participate in nuclear processes, as it has been reported for several organisms [8, 13, 34]. Site-specific proteomic analysis had identified a high proportion of *Tb*SUMO targets involved in DNA replication and repair, chromatin remodeling and transcription, among others [21]. Intriguingly, the characteristic nuclear foci disappeared in chain mutant parasites, which now exhibit a more homogenous distribution in the nucleus as well as in the cytoplasm. These results show that nuclear foci formation relies on polySUMOylation of certain proteins and at the same time confirm the occurrence of SUMO chains in PCFs.

### Bioinformatical detection of SIMs

We next executed a four-step bioinformatic pipeline to predict potential SIMs that could be recruited by poly-SUMO chains. To this end, 11567 trypanosome translated coding sequences were first run against the ScanProsite database (http://prosite.expasy.org/) to search for the three classes of previously published patterns of SIMs, resulting in 1302 protein hits. This output was next filtered to exclude sequences where SIMs reside within reported PFAM domains (214 proteins) or in predicted globular regions (703 proteins). The 385 sequences that survived these criteria were finally evaluated to identify SIMs located in disordered regions yielding a final list of 102 candidate proteins (S1 Table). Regarding the number of SIMs in each protein, 79 sequences have a single motif, 15 sequences have two SIMs, 6 sequences have 3 SIMs, Tb927.9.7690 -one hypothetical protein- contains 4 SIMs and Breast Cancer Type 2 susceptibility protein (BRCA2, Tb927.1.640) harbors 14 SIMs. Near 75% of the SIM candidates are predicted to have nuclear localization according to NucPred server [35] with a NucPred-score >0.5, including a subgroup of 40 proteins displaying the classical eukaryotic nuclear localization signal KRxR that is functional in kinetoplastid parasites. Furthermore, 18 hits were indeed identified as part of the nuclear proteome [36]. Globally, the identified set of SIM hits is enriched in nuclear proteins (chi-square test, p < 0.0002).

**Fig 4:**
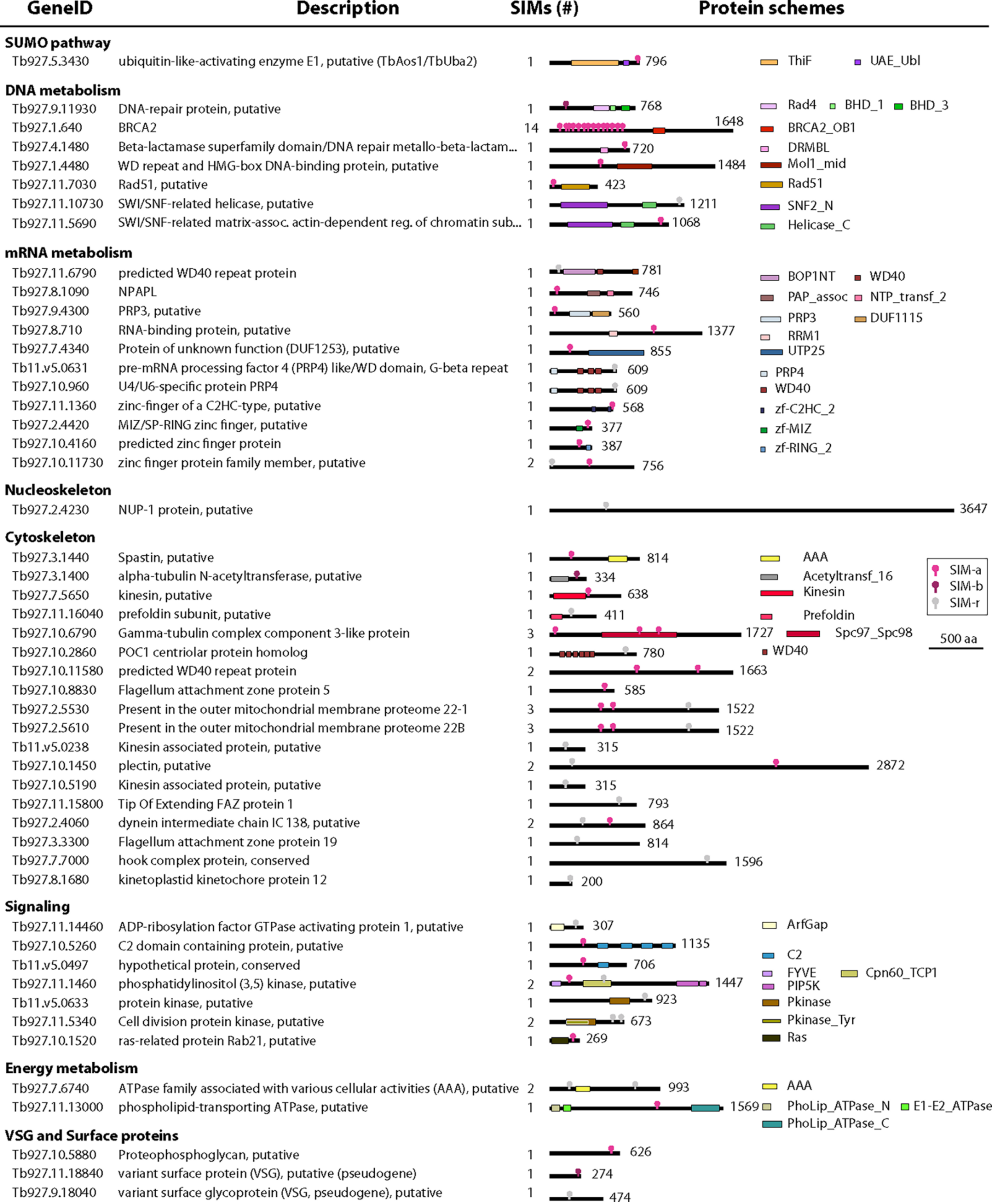
Biological classification of good SIM candidate proteins in *T*. *brucei*. Sequences were identified in the translated proteome using a combination of search methods (see text) and 50 out of 102 hits were classified into functional categories according to their genome annotation or protein domains detected by PFAM database (http://pfam.sanger.ac.uk). GeneID, protein description, total number of SIMs and a scheme of the domains (in scale) are shown. Location of the SIM within each sequence is shown in coloured dot shape: magenta, SIM-a; bourdeux, SIM-b and light gray, SIM-r. Length of the proteins (in residues) are also summarized.

Among the 102 SIM-containing proteins, 43 are hypothetical proteins with unknown functions while 59 proteins have a predicted or experimentally validated function. This latter group was manually classified into functional categories (Fig 4) involving processes often associated with SUMOylation like mRNA metabolism, DNA structure, replication and repair. Gene Ontology enrichment showed that these SIM-containing proteins are related to microtubule cytoskeleton, nuclear lamina, cillium, axonema, nuclear periphery, SUMO activating enzyme complex, among others (S1 Table). Altogether, these results suggest the relevance of the SUMO-associated proteins in nuclear organization in *T*. *brucei*.

### PolySUMOylation and chromatin organization

**Fig 5:**
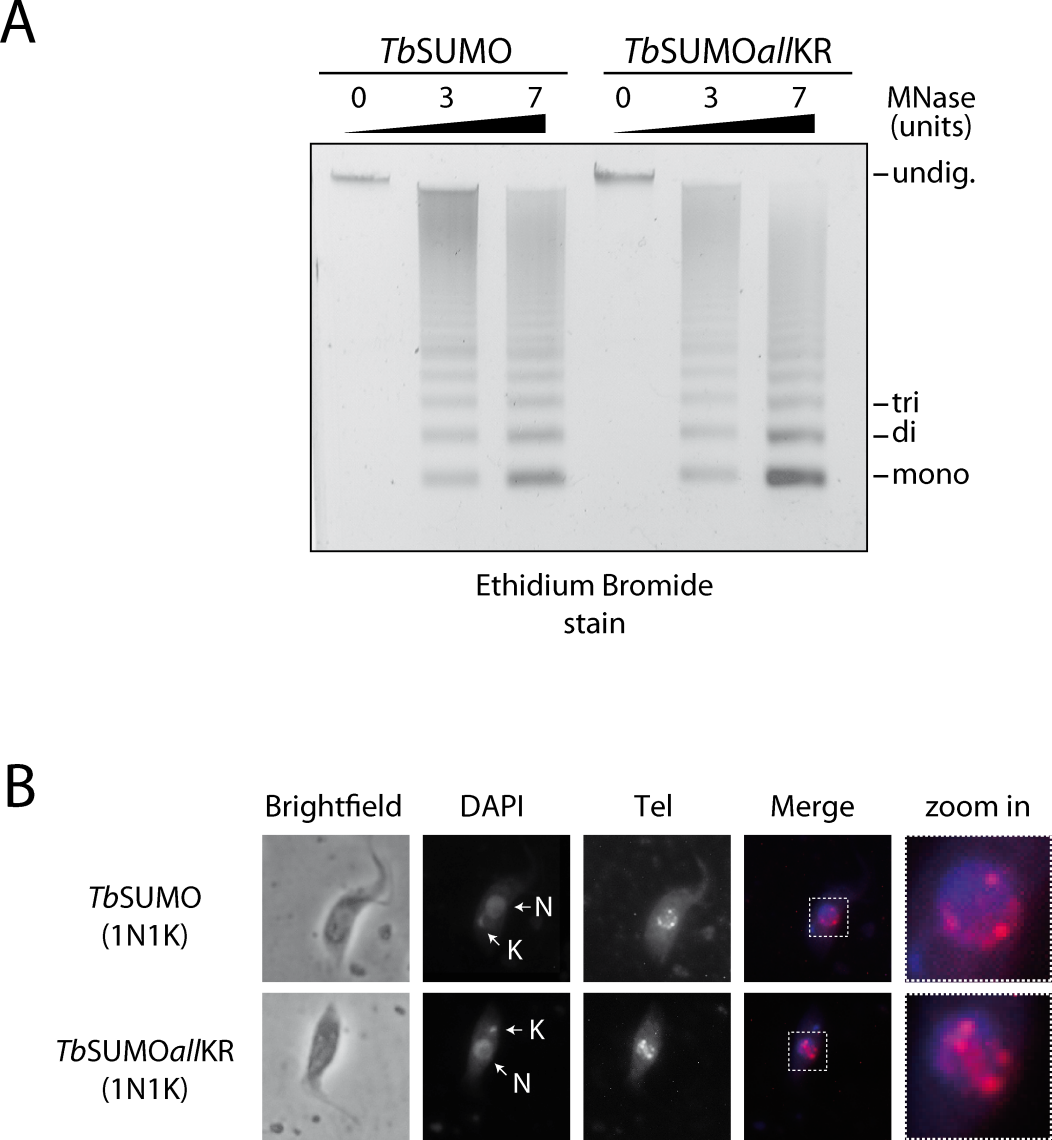
Chromatin organization in *Tb*SUMO*all*KR mutants. **(A)** Genomic DNA isolated from HisHA-*Tb*SUMO (*Tb*SUMO) or HisHA-*Tb*SUMO*all*KR (*Tb*SUMO*all*KR) strains was digested during 5 minutes with increasing units of MNase (0, 3 and 7 U). Samples were separated on 2% agarose gels and visualized under UV light after ethidium bromide staining. Degradation products corresponding to DNA that had been bound to mono-, di-, or tri-nucleosomes, as well as undigested DNA (undig.) are indicated.Bands were quantified with Image Studio software and statistical analysis was performed using Student's t test. **(B)** Fluorescent *in situ* hybridization was used for telomere labelling (Tel, red). Representative images are shown for HisHA-*Tb*SUMO (*Tb*SUMO) and HisHA-*Tb*SUMO*all*KR (*Tb*SUMO*all*KR) parasites. DNA was visualized using DAPI (blue). N, nucleus; K, kinetoplast DNA. Merged images are shown.

Since the group of *T*. *brucei* sequences with predicted SIMs is enriched in nuclear proteins, we sought to test if polySUMOylation could be determinant for the degree of chromatin compactation using the micrococcal nuclease (MNase) digestion assay. As shown in Fig 5A, samples from *Tb*SUMO wild-type or chain mutants showed similar production of a ladder of mono-, di- and tri-nucleosomes, suggesting that SUMO chain formation does not impact on the global chromatin state.

Next, we examined the position of telomeres by fluorescence *in situ* hybridization (FISH) using a probe specific for telomeric repeats. In PCF parasites competent for SUMO chain formation, we observed the typical distribution of telomeres [37]: at the periphery of interphase nuclei (Fig 5B, 1N1K), congregated into a central zone at G2 phase (1N2K) and at opposite poles of dividing nucleus (mitosis) and post-mitotic cells (2N2K) (S4 Fig). Notably, in *Tb*SUMO chain mutants, while no differences were observed for cells in G2/M (S4 Fig), the telomeres of cells in G1/S became clustered and more central with ∼50% of 1N1K cells displaying peripheral localization versus ~70% in the case of control cells (n = 100). Typical patterns are shown in Fig 5B and S4 Fig Altogether, these results suggest that polySUMOylation, although not determinant for chromatin compactation, could participate in telomere positioning.

## Discussion

Previous studies have shown that, like ubiquitin, SUMO is able to form polymeric chains. These chains are involved in several processes, such as mitosis, meiosis and proteosomal degradation of proteins in different organisms [38, 39]. In this work, using a bacterial system designed to express *T*. *brucei* SUMOylation machinery and by site-specific mutagenesis, we were able to determine K27 of *Tb*SUMO as the main residue involved in chain formation.

Mutant PCF parasites unable to form SUMO chains were viable under *in vitro* culture conditions, suggesting that the essential functions of SUMO in *T*. *brucei* can be accomplished in absence of polySUMOylation. Something similar has been reported for *S*. *cerevisiae* where it has been observed that SUMO chains are not essential for vegetative growth but are required for mitosis [40], meiosis [25, 41, 42], DNA replication [43] and repair [44]. Notably, the *all*KR chain mutant parasites showed a less intense pattern of SUMOylation and loss of the *Tb*SUMO nuclear foci normally observed in wild type parasites by indirect immunofluorescence. Taken together these results demonstrate for the first time that SUMO chains are indeed present in *T*. *brucei* PCF, presumably modifying different nuclear proteins as we were not able to detect these structures as free (unanchored) *Tb*SUMO polymers (S5 Fig).

As it has been described in other organisms, SUMO chains could be involved in establishing interaction platforms. For example, these chains are required for the assembly of the synaptonemal complex, a chromosomal structure that links the homologous chromosomes during meiosis. In addition, SUMO chains also participate in the formation of polycomplexes in *S*. *cerevisiae* [25] and particularly in the assembly and function of the complex that catalyzes the resection of double-strand breaks by homologous recombination during vegetative growth and meiosis [44, 45]. In this work, we have identified SIMs in several DNA repair proteins including *Tb*BRCA2, a protein implicated in DNA repair by homologous recombination, with 14 mapped SIMs. Therefore, *Tb*SUMO chains might have a role in DNA repair in *T*. *brucei* as it has been described in yeast.

The characteristic nuclear *Tb*SUMO pattern of PCF cells and the drastic localization change observed in *Tb*SUMO*all*KR parasites (Fig 3D) suggest that polySUMOylation might be regulating the activity of nuclear proteins involved in the formation of subnuclear structures. Similarly, human SUMO-5 polymeric chains have been associated with the enlargement of promyelocytic leukemia nuclear bodies after its ectopic expression [46]. Furthermore, it has been reported that SUMO chains are required for maintenance of chromatin organization and transcriptional repression in *S*. *cerevisiae* [43].

In *T*. *brucei* BSF, a highly SUMOylated focus can be detected at the telomeric VSG expression site exerting a positive regulation [22]. In this work, we have shown that SUMOylation could be important for telomere positioning in PCF, more likely in the case of the abundant minichromosomes. We speculate that abnormal telomere positioning observed in HisHA-*Tb*SUMO*all*KR parasites might be due to the disassembly of protein complexes formed through SUMO-SIM interactions; however, the identification of which factors are involved need to be addressed in future studies.

Considering that SUMOylation is an ubiquitous post-translational modification regulating a wide range of cellular processes, the effects observed in HisHA-*Tb*SUMO*all*KR parasites might be consider as the joint action of a high number of nuclear SUMOylated targets. The processes identified in this work as regulated by polySUMOylation in *T*. *brucei* may constitute the first step to assess the role of these polymeric structures in individual protein targets.

## Materials and methods

### Plasmid constructions

The plasmids CDF-HisHA-*Tb*SUMO were generated as described previously [28]. HisHA-*Tb*SUMO variants were generated by SOEing polymerase chain reaction (PCR). To generate HisHA-*Tb*SUMO K27R and HisHA-*Tb*SUMO*all*KR R27K variants we used plasmid constructions with the complete open reading frame (ORF) of HisHA-*Tb*SUMO or HisHA-*Tb*SUMO with all lysine residues replaced by Arg (GenScript, Piscataway, NJ, USA) as template for SOEing PCR using the following primers: *Tb*SUMO forward CATATGGACGAACACCACC, *Tb*SUMO reverse CTCGAGTCACCCGCCACGCTGCTCCACC, *Tb*SUMOK27R forward GCAGAAGGAACCTGCCGCGAGGAAACTGCACTTG, *Tb*SUMOK27R reverse CAAGTGCAGTTTCCTCGCGGCAGGTTCCTTCTGC, *Tb*SUMOR27K forward GCAGAAGGAACCTGCAAGGAGGAAACTGCACTTG and *Tb*SUMOR27K reverse CAAGTGCAGTTTCCTCCTTGCAGGTTCCTTCTGC. Amplification products were first cloned into pGEM-T Easy vector (Promega, Madison, WI, USA) and sequenced to confirm the presence of the mutated amino acid (Macrogen, Seoul, Korea). Subsequently, *Nde*I/ *Xho*I (New England Biolabs, Ipswich, MA, USA) fragments of HisHA-*Tb*SUMO K27R and HisHA-*Tb*SUMO*all*KR R27K were cloned into the vector pCDFDuet-1-*Tb*E2.

The His tags of the *Tb*E1a and *Tb*E2 enzymes were removed by PCR using the following primers: *Tb*E1a forward CCATGGCCAATGCGGACGAAAAAACG, *Tb*E1a reverse AAGCTTCTACGGGTTGCGCAGGTGCC, *Tb*E2 forward CCATGGCCTCCGGGCTATCTTTAGC and *Tb*E2 reverse GCGGCCGCTTATACCCGCTTCCGGTG. PCR products were cloned into pGEM-T Easy vector (Promega) and sequenced (Macrogen). The *Nco*I*/Hind*III (New England Biolabs) fragment of *Tb*E1a and the *Nco*I/*Not*I (New England Biolabs) fragment of *Tb*E2 were cloned into the vector pACyCDuet-1 and pCDFDuet-1, respectively.

To generate SIMx4 probe we used a synthesized plasmid construction containing the coding sequence for aminoacids 32–132 of RNF4 from *Rattus norvegicus* (GenScript). For mutSIMx4 probe, the same sequence was synthesized but with several mutations: I12A, L14A, V15A, I22A, V23A, L25A, V33A, V24A, V35A, V43A, V44A, I45A and V46A [33, 47]. Both constructions were flanked by *Nhe*I and *Xho*I (New England Biolabs) restriction sites to be cloned in pET28a (Novagen), adding an N-terminal His tag.

### *In bacteria* SUMOylation

*In vivo* reconstituted SUMOylation was performed as described [28]. Briefly, *Escherichia coli* BL21 (DE3) cells were transformed with pCDFDuet-1-*Tb*SUMO/*Tb*SUMO variants-*Tb*E2 and pACYCDuet-1-*Tb*E1a-*Tb*E1b. To assess the SUMOylation reaction cells containing the plasmids mentioned were cultured in Luria–Bertani (LB) medium at 37°C to an OD_600nm_ of 0.6, and then induced with 1 mM isopropyl β-D-1-thiogalactopyranoside (IPTG) (Sigma, Saint Louis, MO, USA) for 5 h at 37°C with vigorous shaking (250 rpm). Cells were then harvested by centrifugation, resuspended in lysis buffer [150 mM NaCl, 50 mM Tris HCl, 0.4 mg/ml lysozyme, 0.1% Triton X-100, 10 mM ethylene diamine tetraacetic acid (EDTA), 1 mM phenylmethylsulfonyl fluoride (PMSF)(Sigma)—pH 7.6] and sonicated when necessary. Samples were then centrifuged for 30 min at 23000 x *g* and 70 μl of supernatants were resuspended in Laemmli sample buffer with 100 mM DTT (7:3) and boiled for 5 min.

### Purification of *Tb*SUMO chains and SIM probes

*E*. *coli* BL21 DE3 competent cells were transformed with plasmids containing the sequence of *Tb*E1, *Tb*E2 and *Tb*SUMO, or with the SIMs probes. Cells were cultured in LB medium at 37°C to an OD_600nm_ of 0.6, and then induced with 1 mM IPTG (Sigma) for 5 h at 37°C with vigorous shaking. Cells were harvested by centrifugation and resuspended in lysis buffer and sonicated. Cleared lysates were obtained by centrifugation at 23000 x *g* for 30 min and subjected to Ni^+2^ chromatography. Briefly, samples were incubated with Ni^+2^-resin (GE Healthcare, Pittsburgh, PA, USA) for 1 h at 4°C with shaking, washed with 30 column volumes (CV) of 50 mM Tris HCl (pH 7.6), 150 mM NaCl, 0.1% Triton X-100, 1mM PMSF and 20 mM imidazole, and bound proteins were eluted with 5 CV of the same buffer but containing 500 mM imidazole. Samples were then resuspended in Laemmli sample buffer with 100 mM DTT (7:3) and boiled for 5 min. Finally, proteins were separated by SDS-PAGE and visualized by Coomassie Blue staining.

### *Tb*SUMO chains pull down *in vitro*

*Tb*SUMO chains or monomer-expressing bacteria were lysed as described previously and then incubated with the SIMx4 probe or the mutSIMx4 probe in 50 mM Tris HCl (pH 7.6), 150 mM NaCl, 1% NP40, 1mM PMSF and 0.5% deoxycholate for 1.5 h at 4°C. Samples were then incubated with Ni^+2^-resin (GE Healthcare) containing 20 mM imidazole for 1 h at 4°C with shaking. Washes were performed with 20 CV of 50 mM Tris HCl (pH 7.6), 250 mM NaCl, 1% NP40 and 1mM PMSF, and with 10 CV of the same buffer but containing 20 mM imidazole. Proteins were eluted with the same buffer but containing 100 mM imidazole (2 CV), 200 mM imidazole (2 CV) or 500 mM imidazole (2 CV), resuspended in Laemmli sample buffer with 100 mM DTT (7:3), boiled for 5 min and separated by SDS-PAGE followed by Western blot analysis using anti-HA antibodies. Purifications from bacteria expressing *Tb*SUMO chains or monomer without the incubation with the probes were performed as control.

### *Tb*SUMO chains pull down *in vivo*

About 5 x 10^8^ *T*. *brucei brucei* 427 PCF parasites [48] and 25 ml of induced cultures of SIMs probes were collected by centrifugation, resuspended in 1.25 ml of lysis buffer (500 mM NaCl, 50 mM Tris-HCl, 1 mM PMSF, 1% NP40, 20 mM N-ethylmaleimide (NEM) (Sigma)- pH 7.5) and sonicated up to loss of viscosity. For further purification lysates were cleared by centrifugation for 15 min at 23000 x *g* at 4°C and supernatants were incubated with each other for 1.5 h at 4°C with gentle stirring, adding 0.5% deoxycholate. Samples were then subjected to Ni^+2^ chromatography as described above. Purifications from parasites without incubation with SIMs probes were used as control.

### Trypanosome culture

In this work we used the PCF of *T*. *brucei brucei* Lister 427 [48]. As describe previously, parasites were cultured at 28°C in SDM-79 medium [49] (Life Technologies, Carlsbad, CA, USA) supplemented with 10% (vol/vol) heat-inactivated fetal calf serum (Natocor, Córdoba, Argentina) and 7.5 mg/l hemin. Parasites viability was evaluated by growth curves obtained by counting cell number daily [21].

### Generation of *Tb*SUMO-transfectant cell lines

For HisHA-*Tb*SUMO strain, a HisHA-*Tb*SUMO construct was synthesized (GenScript), containing the coding sequence for an 8His (24bp)-HA (27bp) tag, 249bp of the 5'end of *Tb*SUMO open reading frame (ORF; Tb927.5.3210), a *Xho*I restriction site and 250bp of the 3'end of 5'untranslated region (UTR) of *Tb*SUMO. For HisHA-*Tb*SUMO*all*KR strain, the former construction was modified by replacing the 5'end of *Tb*SUMO ORF by the complete *Tb*SUMO ORF with all 8 lysine residues replaced by arginine residues and 200bp of the 5'end of 3'UTR of this gen (GenScript). These constructions were flanked by *Hind*III and *Bam*HI restriction sites to be cloned in pEnT6P, an endogenous locus tagging vector containing a puromycin or hygromycin resistance marker cassette, for sequential replacement of both *Tb*SUMO alleles [50]. The vectors were linearized and electroporated as described previously [21]. Transfected cells were cloned by limiting dilution and selected with 2 μg/ml of puromycin or 25 μg/ml of hygromycin (InvivoGen, San Diego, CA, USA) in 96-wells plates as described in http://tryps.rockefeller.edu/. To confirm the appropriated replacement of endogenous *Tb*SUMO allele we performed a PCR using a sense primer corresponding to the upstream gene Tb927.5.3220 of *Tb*SUMO in the genome (Up foward: GTGACTCGTTTGTACCTCAC) and an antisense primer corresponding to pEnT6P puromycin (Puro reverse: CGTGGGCTTGTACTCGGTC) or hygromycin (Hygro reverse: GCCTATTCCTTTGCCCTCGG). The absence of endogenous *Tb*SUMO after the replacement of both alleles was confirmed by PCR using a sense primer corresponding to the 5'UTR of *Tb*SUMO (5'UTR foward: GAGTTGGGTTCATTTCTGAGCC) and an antisense primer corresponding to the 3'UTR of *Tb*SUMO (3'UTR reverse: AGTTGTGATGGACGAAGCAG).

To assess the ability of *Tb*SUMO to form free chains *in vivo* we generated a *Tb*SUMO variant unable to modified targets, by removing the di-glycine motif (ΔGG), but capable of being modified by other *Tb*SUMO molecules, due to the presence of acceptor lysines. As negative control we generated a *Tb*SUMO variant which was deficient in the di-glycine motif and the acceptor lysines. To generate *Tb*SUMOΔGG-3xFlag and *Tb*SUMO*all*KRΔGG-3xFlag variants we used plasmid constructions with the complete ORF of *Tb*SUMO or *Tb*SUMO*all*KR as template for PCR using the following primers: *Tb*SUMO forward CATATGGACGAACACCACC, *Tb*SUMO reverse CTCGAGTCACCCGCCACGCTGCTCCACC, *Tb*SUMOΔGG forward GCGGGATCCATGGACGAACCCACTCATAACTCCAACAACG, *Tb*SUMOΔGG reverse GGCTCTAGATGTCTGCTCCACCATCGCATCAATC. Amplification products were first cloned into pGEM-T Easy vector (Promega) and sequenced (Macrogen), after which they were digested with *BamH*I (New England Biolabs) and *Xba*I (New England Biolabs) and cloned into the vector pRP^TAG^, modified to add a 3xFlag tag at the N-terminal or C-terminal end of the construction. The vectors were linearized and electroporated into PCF *T*. *brucei brucei* Lister 427-pLew13 as described above. Transfected parasites were induced with 5 μg/ml of doxycycline (Sigma) for 48 h.

### Electrophoresis and immunoblotting

Proteins were separated by SDS-PAGE (10% acrylamide or 7.5%-12.5% discontinuous acrylamide) followed by Coomassie Blue staining or transferred to a nitrocellulose Hybond ECL membrane (GE Healthcare). Membranes were incubated with high-affinity rat monoclonal antibodies anti-HA (Roche, Basel, Switzerland) diluted 1:1000, mouse monoclonal antibodies anti-Flag M2 (Sigma) diluted 1:5000, mouse monoclonal antibodies anti-polyHistidine diluted 1:250 (Sigma), mouse monoclonal antibodies anti-α-tubulin (Sigma) diluted 1:5000, rabbit antibodies anti-*Tc*SUMO diluted 1:500 or rabbit antibodies anti-*Tb*SUMO diluted 1:500, followed by detection by goat anti-rat, anti-mouse or anti-rabbit Alexa Fluor® 790 or 680 (Jackson, West Grove, PA, USA) diluted 1:25000 using an Odyssey CLx Infrared Imaging System (LI-COR Biosciences, Lincoln, NE, USA). Bands of three replicates were quantified with Image Studio software and statistical analysis was performed using SPSS Statistics software and tests described for each case. Prestained Protein Molecular Weight markers used were from Pierce (Rockford, IL, USA).

### Indirect immunofluorescence

PCF parasites were subjected to inmunofluorescence as previously described [21]. The anti-HA antibody (Roche) and the anti-*Tb*SUMO antibody were used at a 1:500 dilution, while secondary antibodies were used at a 1:1000 dilution [polyclonal goat anti-rat Alexa Fluor^®^ 546 or polyclonal goat anti-mouse Alexa Fluor^®^ 488 (Jackson)]. Finally, images were obtained with a fluorescence microscope Nikon 80i LED and analyzed by ImageJ software.

### *In situ* hybridization

This protocol was carried out as described by the Cross laboratory (http://tryps.rockefeller.edu/), with some modifications. Briefly, 1×10^6^ parasites were washed with PBS and fixed in 4% paraformaldehyde for 10 min at room temperature. After two washes with PBS the cells were attached onto poly-lysine coated glass coverslips for 30 min and incubated with 25 mM NH_4_Cl for 15 min. Parasites were permeabilized with 0.2% saponin in PBS for 30 min at room temperature. The DNA was denatured by incubating permeabilized cells in 70% formamide/2×SSC for 5 min at 70°C. The coverslips were washed briefly in 2×SSC and blocked for 3 h in hybridization mix (SSC 4x, Denhardt 5X, 8 μg/ml yeast tRNA, 8 μg/ml herring sperm DNA, 5% dextran sulfate, 60% formamide). Parasites were then probed with a Cy3-labeled (CCCTAA)_9_ probe (Macrogen) at 50°C, overnight. Coverslips were then washed with SSC 4x-40% formamide for 10 min, SSC 4x for 10 min, SSC 2x for 10 min and two times with SSC 1x for 5 min. Finally, coverslips were mounted using FluorSave reagent (Merck) containing 5 mg/ml DAPI (Life Technologies). Images were obtained with a fluorescence microscope Nikon 80i LED and analyzed by ImageJ software.

### Micrococcal nuclease digestion

Micrococcal assay was adapted from [51] and [52]. Parasites (5x10^7^) were washed in a buffer containing 1 mM L-glutamate, 250 mM sucrose, 2.5 mM CaCl_2_ and 1 mM PMSF, and then lysed with the same buffer but also containing 1% Triton X-100. Pellets were washed [10 mM HEPES, 35 mM NaCl, 500 μM MgCl_2_, 500 μM CaCl_2_, 1 mM PMSF, 5.2 mM β-mercaptoethanol (Sigma)] and incubated with increasing amounts of microccocal nuclease (New England Biolabs) for 5 min at 37°C (0, 3, 7 U). Reactions were stopped by adding a stop solution (0.09 M EDTA, 0.72% SDS). Extractions were performed with chloroform and DNA was precipitated with isopropanol. Finally, the DNA was washed with 70% ethanol, resuspended in a buffer containg 50 mM Tris HCl pH 8 and 10 mM EDTA and incubated with 10 μg/ml of RNAse for 2 h. Samples were electrophoresed on horizontal 2% agarose gels and stained with ethidium bromide. Bands were quantified with Image Studio software and statistical analysis was performed using Student's t test.

### Identification of good SIM candidates in *Trypanosoma brucei* genome

We performed an *in silico* search with four different evaluation criteria to priorizate good candidate proteins containing multiple SIMs that could be recruited by poly-SUMO chains. Briefly, we generated a FASTA-formatted version of the translated *T*. *brucei* coding sequences downloaded from TriTrypDB v9.0 [URL www.tritrypdb.org [53]] and used the ScanProsite server [URL http://www.expasy.org/tools/scanprosite [54]] to screen the entire proteome of species for the presence of the three classes of previously published SIMs: SIM-a (PILVM)-(ILVM)-x-(ILVM)- (DSE>)(3); SIM-b (PILVM)-(ILVM)-D-L-T; and SIM-r (DSE)(3)-(ILVM)-x-(ILVMF)(2). The hits reported in the previous step were further validated using PFAM server to search for homology domains [URL http://pfam.xfam.org [55]], and all SIM candidates located within a known domain were filtered. Next, we run IUPRED program [URL http://iupred.enzim.hu/ [56]] with the "structured regions" option to evaluate if SIM candidates reside in a region predicted as globular (much less likely to be a functional SUMO binder). Output hits that pass these filtering criteria were finally evaluated using the same software for disorder prediction (with the option "long disorder" and cut-off value above 0.5), since if the SIM candidate resides in a region predicted to be disordered increases its chances to be functional. GO enrichment analysis was performed using Tritrypdb Analyze Results tool (http://tritrypdb.org) with Fisher exact test filtering for false discovery rate (FDR) lower than 0.1.

## Supporting information

S1 Fig SUMOylation of *Tb*E1a and *Tb*E2. *E*. *coli* BL21 DE3 cells expressing *Tb*E1a-*Tb*E1b, His-*Tb*E2 and *Tb*SUMO (complete system His-*Tb*E2) or *Tb*E2, His-*Tb*E1a-*Tb*E1b and *Tb*SUMO (complete system His-*Tb*E1a) were lysed and cleared lysates were subjected to Ni^+2^ chromatography. Proteins were separated by SDS-PAGE and visualized by Western blot using anti-His antibodies. Thus, it was possible to observe His-*Tb*E2 (~30 kDa) and higher molecular weight bands corresponding to SUMOylated *Tb*E2. Also, we visualized His-*Tb*E1a (~40 kDa) and high molecular weight bands corresponding to SUMOylated *Tb*E1a.
(TIF)

S2 Fig Poly SUMO-SIM binding controls and *Tb*SUMO monomer assay. **(A)** *E*. *coli* BL21 DE3 cells expressing HA-*Tb*SUMO chains (I: Input) were incubated with Ni^+2^-Sepharose beads without adding the SIMx4 or the mutSIMx4 probes (as a negative control, P.D.: pull-down). Proteins present in the input and the eluate were visualized by Western blot using anti-HA antibodies. **(B)** Quantification of poly-SUMO chains binding to the probes. **(C)** *E*. *coli* BL21 DE3 cells expressing HA-*Tb*SUMO monomer (I: Inputs) were incubated with the SIMx4 probe, the mutSIMx4 probe or with resin (negative control), and pulled-down (P.D.) using Ni^+2^-Sepharose beads. Proteins present in the inputs and in the eluates were visualized by Western blot using anti-HA antibodies. **(D)** Quantification of *Tb*SUMO monomer binding to the probes. **(E)** Cell free extract from *T*. *brucei* 427 PCF (I: Input) was incubated with Ni^+2^-Sepharose beads without adding the SIMx4 or the mutSIMx4 probes (as a negative control, P.D.: pull-down). Proteins present in the input and eluates were visualized by Western blot using anti-*Tb*SUMO antibodies, and anti-tubulin antibody was used as the loading control for the input. **(F)** Quantification of poly-SUMO conjugates produced in *T*. *brucei* parasites that bind to the probes. n.d., not detected. All experiments in B, D and F were done in triplicate, bands in each lane were quantified with Image Studio software and statistical significance was determined using one-way ANOVA with Bonferroni post hoc test for multiple comparisons. Brackets denote significant differences between the indicated groups (*,p<0.01).
(TIF)

S3 Fig Generation of HisHA-*Tb*SUMO*all*KR cell line. **(A)** Schematic representation of genomic locus of wild type, single transfectant and double transfectant parasites (*Tb*SUMO*all*KR). Primers used to confirm the replacement of the wild-type alleles are shown. **(B)** Ethidium bromide-stained agarose gel of PCR products confirming the appropriate replacement of endogenous *Tb*SUMO alleles.
(TIF)

S4 Fig Telomeres localization in *Tb*SUMO*all*KR mutants. Fluorescent *in situ* hybridization was used for telomere labelling (Tel, red). The insets of representative images of different phases of the cell cycle are shown for HisHA-*Tb*SUMO **(A)** and HisHA-*Tb*SUMO*all*KR **(B)** parasites. DNA was visualized using DAPI (blue). Merged images are shown.
(TIF)

S5 Fig Formation of free SUMO chains in *T*. *brucei*. PCF parasites were transfected with wild type *Tb*SUMO or *Tb*SUMO*all*KR variants lacking the diGlycine motif (ΔGG), fused to tri-Flag epitope at the C-terminus (*Tb*SUMO ΔGG and *Tb*SUMO*all*KR ΔGG, respectively). These *Tb*SUMO variants cannot be attached to other proteins but can be substrates of endogenous *Tb*SUMO. **(A)** Growth curves of parasites with induced overexpression of *Tb*SUMO ΔGG or *Tb*SUMO*all*KR ΔGG (dox +) compared to uninduced parasites (dox -). **(B)** Parasites extracts were analyzed by Western blot using anti-Flag antibodies to assess the presence of high molecular weight bands compatible with *Tb*SUMO free polymers. The asterisk denotes a crossreacting band. **(C)** Immunofluorescence analysis of *Tb*SUMO ΔGG or *Tb*SUMO*all*KR ΔGG parasites using anti-Flag antibodies. Nuclear and kinetoplast DNA were visualized by DAPI staining (blue). Representative images of anti-Flag-DAPI merged images are shown.
(TIF)

S1 Table List of good SIM candidates. Table showing the following columns: GeneID, Description, numbers of SIM per protein, location of SIM1 within the protein sequence (start, end), type of SIM and sequence, NucPred score, location of SIM in low complexity region and pfam domains.
(XLSX)
