# Supplementary figures and images for: SUMO polymeric chains are involved in nuclear foci formation and chromatin organization in Trypanosoma brucei procyclic forms
Source: PLoS One. 2018 Feb 23;13(2):e0193528. doi: 10.1371/journal.pone.0193528 (PMC5825156; doi:10.1371/journal.pone.0193528)

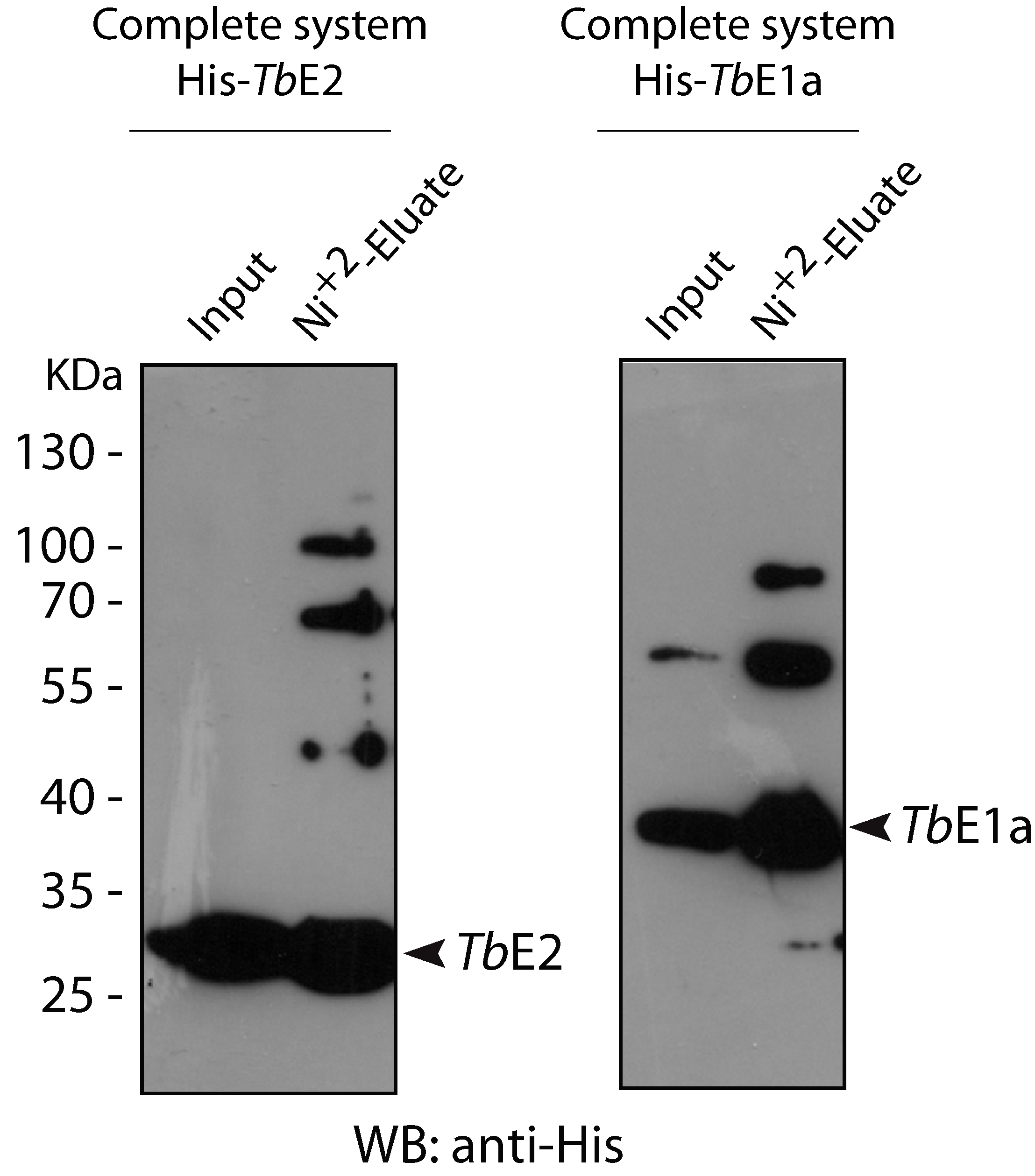

Supplement: S1 Fig — E. coli BL21 DE3 cells expressing TbE1a-TbE1b, His-TbE2 and TbSUMO (complete system His-TbE2) or TbE2, His-TbE1a-TbE1b and TbSUMO (complete system His-TbE1a) were lysed and cleared lysates were subjected to Ni+2 chromatography. Proteins were separated by SDS-PAGE and visualized by Western blot using anti-His antibodies. Thus, it was possible to observe His-TbE2 (~30 kDa) and higher molecular weight bands corresponding to SUMOylated TbE2. Also, we visualized His-TbE1a (~40 kDa) and high molecular weight bands corresponding to SUMOylated TbE1a. (TIF) [file pone.0193528.s001.tif]

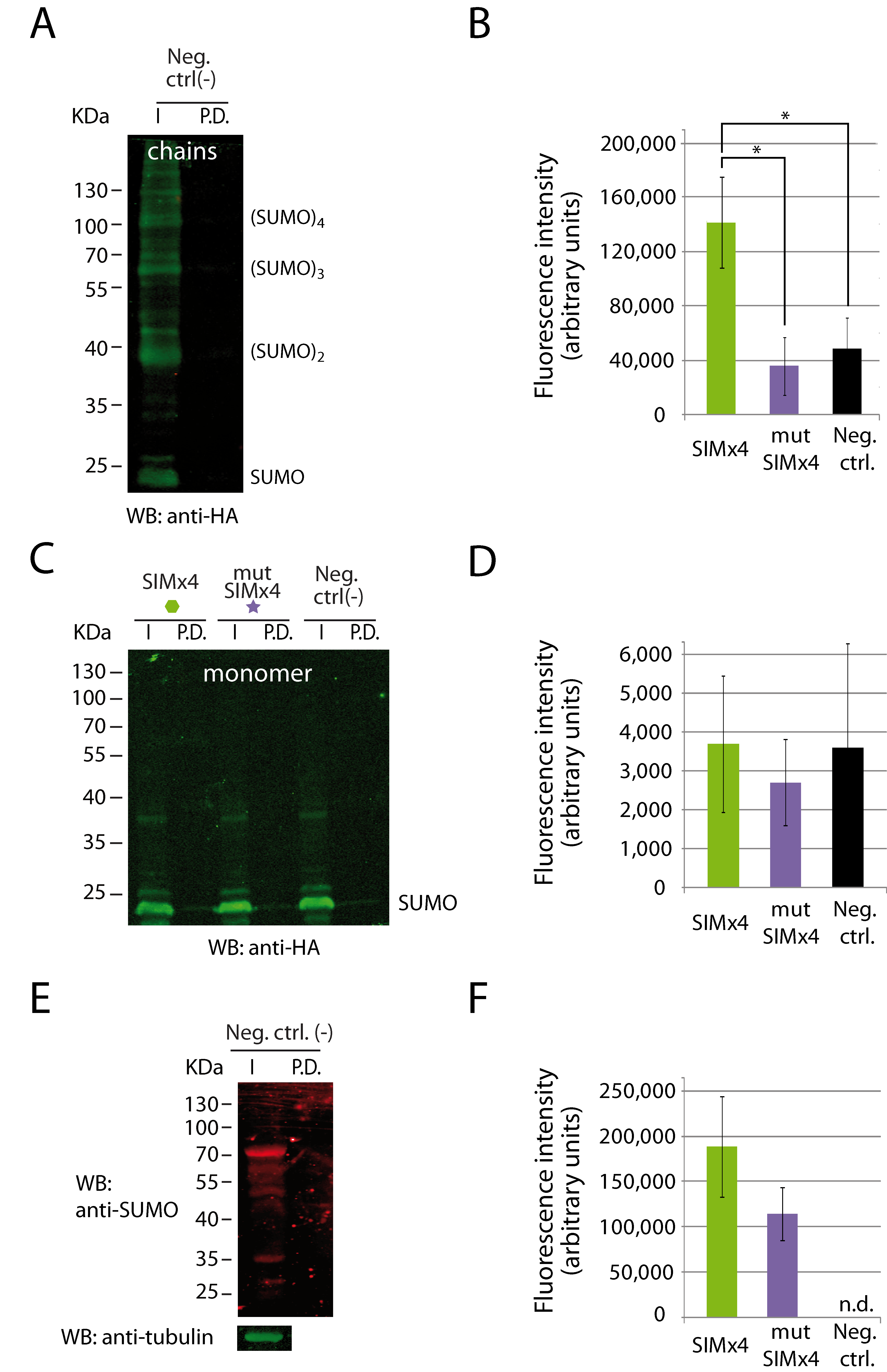

Supplement: S2 Fig — (A) E. coli BL21 DE3 cells expressing HA-TbSUMO chains (I: Input) were incubated with Ni+2-Sepharose beads without adding the SIMx4 or the mutSIMx4 probes (as a negative control, P.D.: pull-down). Proteins present in the input and the eluate were visualized by Western blot using anti-HA antibodies. (B) Quantification of poly-SUMO chains binding to the probes. (C) E. coli BL21 DE3 cells expressing HA-TbSUMO monomer (I: Inputs) were incubated with the SIMx4 probe, the mutSIMx4 probe or with resin (negative control), and pulled-down (P.D.) using Ni+2-Sepharose beads. Proteins present in the inputs and in the eluates were visualized by Western blot using anti-HA antibodies. (D) Quantification of TbSUMO monomer binding to the probes. (E) Cell free extract from T. brucei 427 PCF (I: Input) was incubated with Ni+2-Sepharose beads without adding the SIMx4 or the mutSIMx4 probes (as a negative control, P.D.: pull-down). Proteins present in the input and eluates were visualized by Western blot using anti-TbSUMO antibodies, and anti-tubulin antibody was used as the loading control for the input. (F) Quantification of poly-SUMO conjugates produced in T. brucei parasites that bind to the probes. n.d., not detected. All experiments in B, D and F were done in triplicate, bands in each lane were quantified with Image Studio software and statistical significance was determined using one-way ANOVA with Bonferroni post hoc test for multiple comparisons. Brackets denote significant differences between the indicated groups (*,p<0.01). (TIF) [file pone.0193528.s002.tif]

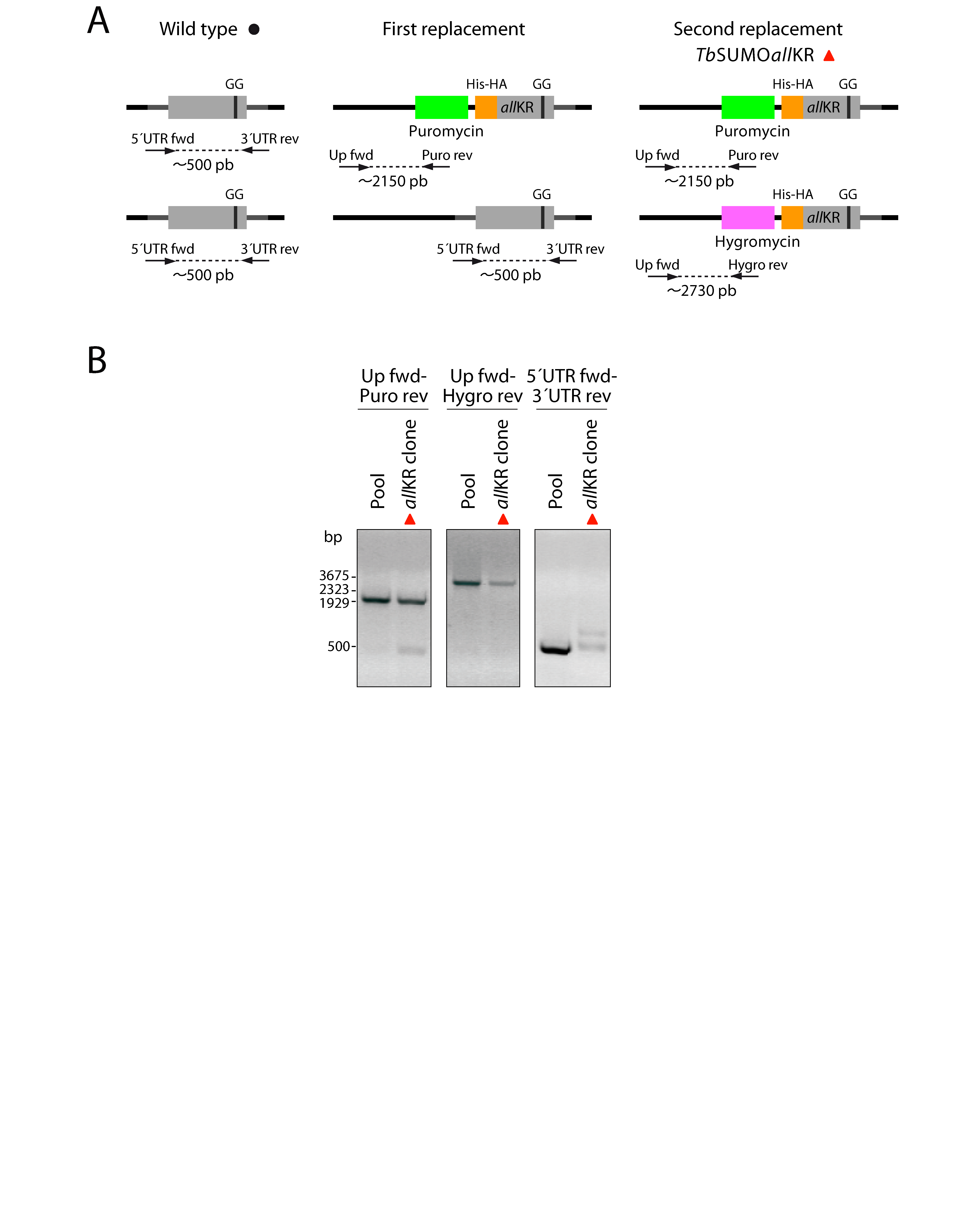

Supplement: S3 Fig — (A) Schematic representation of genomic locus of wild type, single transfectant and double transfectant parasites (TbSUMOallKR). Primers used to confirm the replacement of the wild-type alleles are shown. (B) Ethidium bromide-stained agarose gel of PCR products confirming the appropriate replacement of endogenous TbSUMO alleles. (TIF) [file pone.0193528.s003.tif]

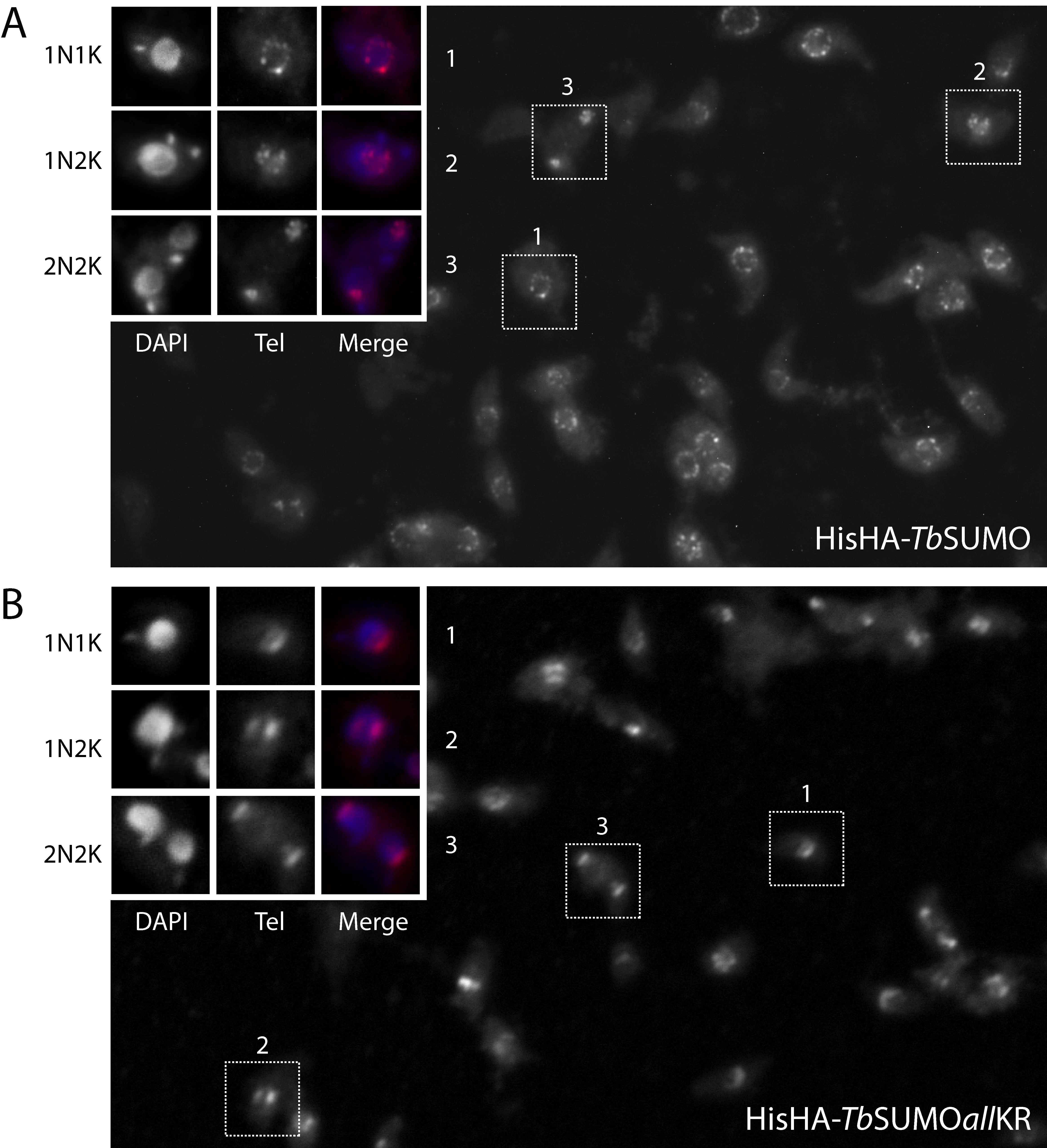

Supplement: S4 Fig — Fluorescent in situ hybridization was used for telomere labelling (Tel, red). The insets of representative images of different phases of the cell cycle are shown for HisHA-TbSUMO (A) and HisHA-TbSUMOallKR (B) parasites. DNA was visualized using DAPI (blue). Merged images are shown. (TIF) [file pone.0193528.s004.tif]

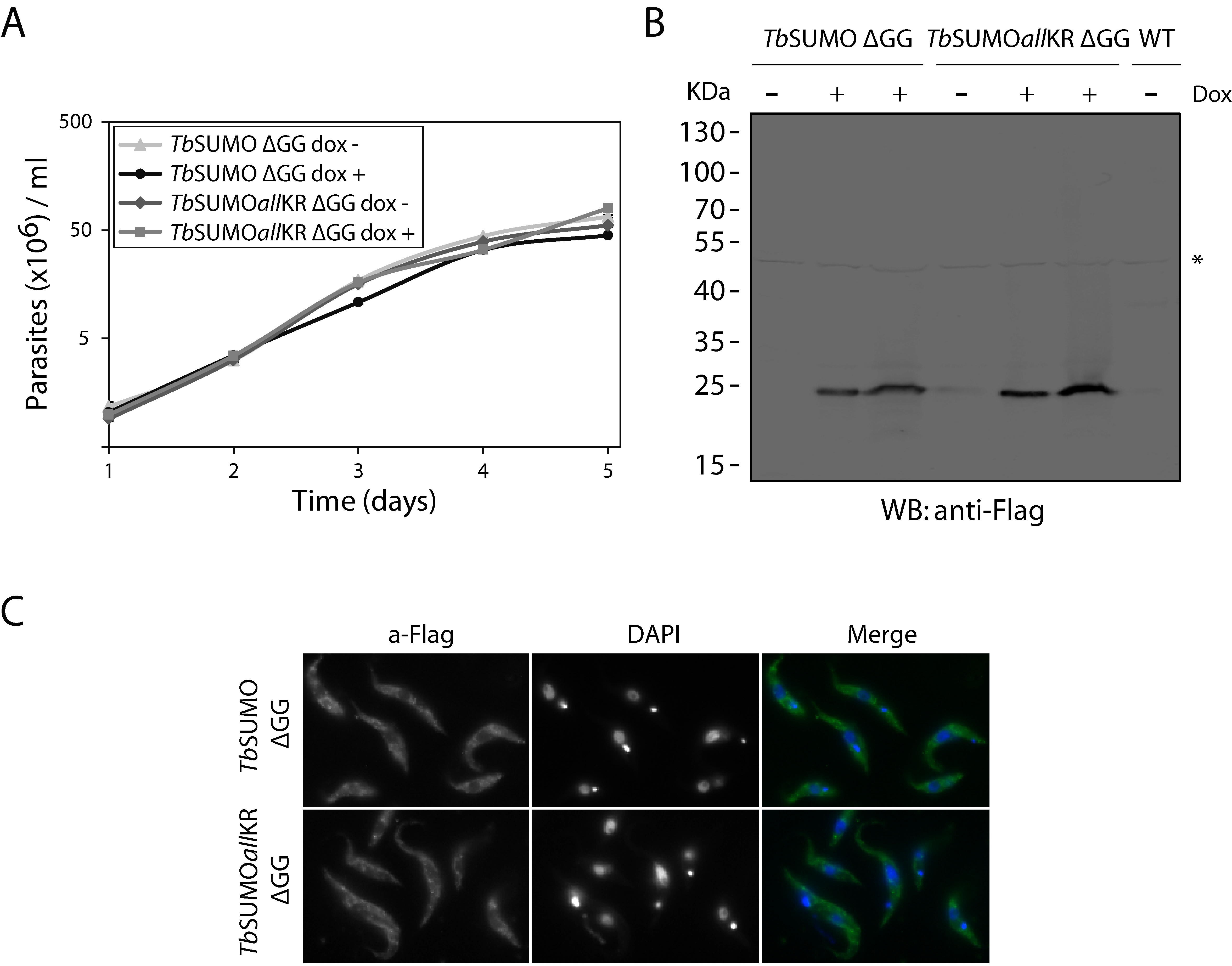

Supplement: S5 Fig — PCF parasites were transfected with wild type TbSUMO or TbSUMOallKR variants lacking the diGlycine motif (ΔGG), fused to tri-Flag epitope at the C-terminus (TbSUMO ΔGG and TbSUMOallKR ΔGG, respectively). These TbSUMO variants cannot be attached to other proteins but can be substrates of endogenous TbSUMO. (A) Growth curves of parasites with induced overexpression of TbSUMO ΔGG or TbSUMOallKR ΔGG (dox +) compared to uninduced parasites (dox -). (B) Parasites extracts were analyzed by Western blot using anti-Flag antibodies to assess the presence of high molecular weight bands compatible with TbSUMO free polymers. The asterisk denotes a crossreacting band. (C) Immunofluorescence analysis of TbSUMO ΔGG or TbSUMOallKR ΔGG parasites using anti-Flag antibodies. Nuclear and kinetoplast DNA were visualized by DAPI staining (blue). Representative images of anti-Flag-DAPI merged images are shown. (TIF) [file pone.0193528.s005.tif]
